# Balanced Trade-Offs between Alternative Strategies Shape the Response of *C. elegans* Reproduction to Chronic Heat Stress

**DOI:** 10.1371/journal.pone.0105513

**Published:** 2014-08-28

**Authors:** Erin Z. Aprison, Ilya Ruvinsky

**Affiliations:** 1 Department of Ecology and Evolution and Institute for Genomics and Systems Biology, The University of Chicago, Chicago, Illinois, United States of America; 2 Department of Organismal Biology and Anatomy, The University of Chicago, Chicago, Illinois, United States of America; CSIR-Central Drug Research Institute, India

## Abstract

To ensure long-term reproductive success organisms have to cope with harsh environmental extremes. A reproductive strategy that simply maximizes offspring production is likely to be disadvantageous because it could lead to a catastrophic loss of fecundity under unfavorable conditions. To understand how an appropriate balance is achieved, we investigated reproductive performance of *C. elegans* under conditions of chronic heat stress. We found that following even prolonged exposure to temperatures at which none of the offspring survive, worms could recover and resume reproduction. The likelihood of producing viable offspring falls precipitously after exposure to temperatures greater than 28**°**C primarily due to sperm damage. Surprisingly, we found that worms that experienced higher temperatures can recover considerably better, provided they did not initiate ovulation. Therefore mechanisms controlling this process must play a crucial role in determining the probability of recovery. We show, however, that suppressing ovulation is only beneficial under relatively long stresses, whereas it is a disadvantageous strategy under shorter stresses of the same intensity. This is because the benefit of shutting down egg laying, and thus protecting the reproductive system, is negated by the cost associated with implementing this strategy – it takes considerable time to recover and produce offspring. We interpret these balanced trade-offs as a dynamic response of the *C. elegans* reproductive system to stress and an adaptation to life in variable and unpredictable conditions.

## Introduction

To be successful in dynamic environments, organisms must have ways of buffering the effects of changes in their surroundings. These changes can have regular periodicity, such as seasonal variation and circadian oscillations, or they can be irregular environmental fluctuations. Temperature is a ubiquitous variable. Therefore, systems that ameliorate the effects of thermal stress are critical, particularly in plants and small ectotherms that cannot easily avoid deleterious temperatures [1]. When change is frequent or prefaced by reliable environmental cues, phenotypic plasticity – the sum of adaptive responses to change, is favored [2]. When environmental fluctuations are infrequent and potentially catastrophic, some organisms employ bet-hedging strategies to ensure the survival of a fraction of their offspring during extreme conditions [3]–[6].

One well-described strategy for dealing with environmental extremes in an animal is the formation of resistant dauer larvae in *Caenorhabditis elegans*. Instead of developing into reproductive adults, worms exposed to higher temperatures [7] or other unfavorable environments [8], [9] arrest as persistent larvae [10]. However, worms can only enter the dauer stage during a limited time window late in the L1 larval period [11], [12]. Expecting that animals of other developmental stages have different strategies for coping with adverse environments, we investigated the response of adults to high temperature.

When challenged by a sudden rise in temperature, *C. elegans* can mount a robust heat shock response [13]. However, this response becomes attenuated over a period of hours [14], [15], making it unclear what mechanisms worms use to cope with longer durations of stress. Moreover, accumulation of heat shock proteins can be detrimental [16]. Exposure to mild heat stress (temperatures of 28**°**C and above) has been reported to cause sterility in *C.* *elegans* [17]–[19] and reproduction is particularly sensitive to thermal stress, with brood size decreasing with increasing temperature [20], [21]. Despite this apparent fragility, strains of *C.* *elegans* have been found across the globe at sites where fluctuating temperatures routinely exceed 27**°**C [22]–[24] and the temperatures inside rotting fruits, thought to be a natural habitat of *C.* *elegans* [23], [25], [26] can be even higher [27]. To understand how this can be achieved, we investigated reproductive performance of *C.* *elegans* hermaphrodites during and in recovery from chronic heat stress.

## Results

### *C. elegans* can recover fecundity after chronic heat stress

**Figure 1:**
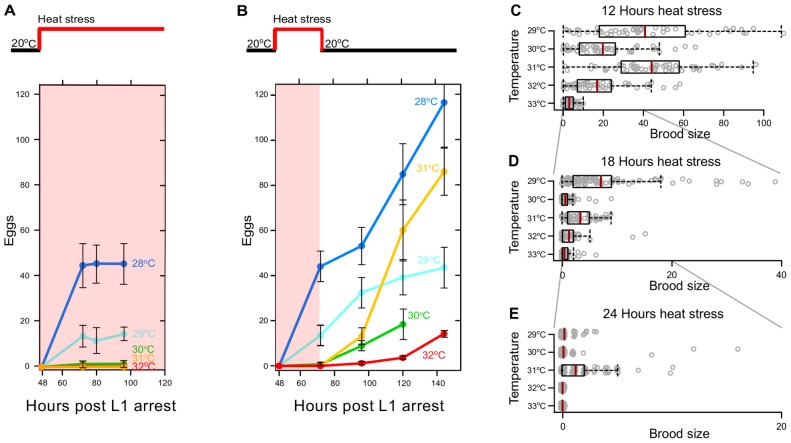
Hermaphrodites can recover egg laying after 24 hours of heat stress. (A) Average numbers of eggs laid by individual hermaphrodites when shifted (from 20°C) to stress temperatures between 28°C and 32°C. Note that at 20°C worms lay ∼300 eggs. (B) Average numbers of eggs laid by individual hermaphrodites that were shifted back down to 20**°**C after 24 hours of heat stress. Error bars represent s.d. Brood sizes produced by individual hermaphrodites stressed for (C) 12, (D) 18, and (E) 24 hours. Each gray circle represents one animal. Vertical red lines on box plots represent means.

*C. elegans* reproduction is exquisitely sensitive to temperature [20], [21]. When raised at 20**°**C, individual worms produce an average of approximately 300 offspring. We investigated reproductive performance at a variety of temperatures. In some experiments, we counted all deposited “eggs” (without ascertaining whether they have been fertilized or their viability). In other experiments, we traced the fate of laid eggs beyond hatching and refer to them as “embryos”. When young adult worms raised at 20**°**C were shifted to higher temperatures (28**°** to 32**°**C, Figure 1A), they laid very few eggs during the first 24 hours of heat stress and none during continued stress. At temperatures of 31**°**C and above, worms did not lay any eggs. In these and other experiments described below, tested worms were singled as young adults to exclude possible effects of density on a variety of phenotypes (see Materials and Methods for details of experimental design).

To test whether exposure to higher temperatures caused irreversible sterility, worms that were raised at 20**°**C were shifted to higher temperatures for 24 hours (oviposition during heat stress reaches a plateau in under 24 hours) and then shifted back down to 20**°**C to recover. Worms treated in this way regained the ability to lay additional eggs (Figure 1B). This is true even for temperatures above 30**°**C at which no eggs were laid during stress. Surprisingly worms that were shifted to 31**°**C, laid more eggs upon recovery than worms that were shifted to 29**°**C or 30**°**C.

For recovery to be meaningful, adult worms have to survive periods of chronic heat stress and produce embryos that hatch into viable progeny. Producing even a single viable offspring may be sufficient, since *C. elegans* are self-fertile hermaphrodites. To test whether eggs, laid during stress or after recovery, develop into viable larvae, we systematically subjected individual young adult hermaphrodites to periods of heat stress of different intensities and durations and allowed them to recover. We chose durations of 12, 18, 24, and 36 hours–periods of heat stress much longer (∼100 fold) than the typical heat shock [28]–[31]. Individual worms were monitored during five days of recovery at 20**°**C for production of larvae. None of the embryos laid at 30**°**C were able to hatch and only a few hatched at 29**°**C. Remarkably, hermaphrodites subjected to these and higher temperatures did nevertheless recoup some fecundity (Figure 1C–E) when they were shifted down to the permissive temperature (20**°**C). Two trends stood out: worms treated with shorter durations of heat stress appeared to have been less damaged since they produced larger brood sizes and worms subjected to heat stress at 31**°**C recovered better than expected – they had brood sizes similar to worms exposed to 29**°**C. Nearly all of the worms that had been heat-stressed for 12 hours at a range of temperatures from 29**°**C to 33**°**C were able to recover and produce viable offspring (Figure 1C), whereas there was no recovery after 36 hours of stress (Table S1, Table S2). After 24 hours of stress, the only group that recovered a brood size larger than an average of one was that of worms that had been at 31**°**C (Figure 1E, Figure S1).

### Latency in the egg laying system

**Figure 2:**
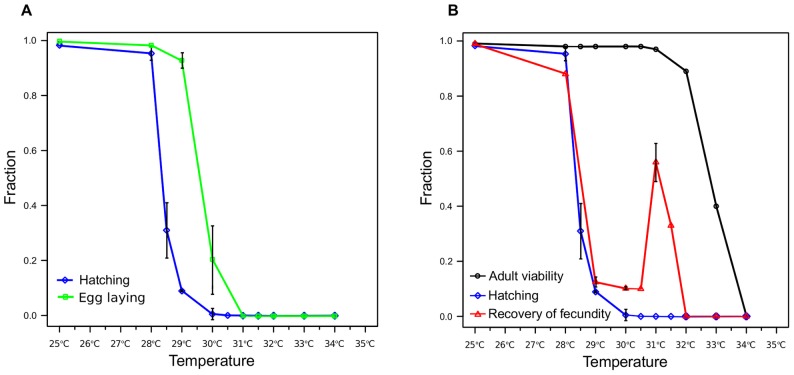
Reproduction across a range of chronic stress temperatures. (A) Fractions of embryos laid at 20°C that hatch after 24 hours at a given stress temperature (blue) and fraction of adult worms that lay eggs at a given temperature (green). (B) In addition to the same hatching curve as in A, fractions of worms that recover fecundity following 24 hours at a given stress temperature (red) and adult viability after 24 hours (black) are shown. Error bars represent s.d.

After noting that a small number of embryos could hatch at 29**°**C, we performed systematic experiments to test the ability of embryos to hatch during chronic heat stress (Figure 2A). At temperatures up to 28**°**C most embryos withstood 24 hours of heat stress, whereas at 30**°**C only ∼1% did. We expected the fraction of hermaphrodites that continued to lay eggs during heat stress to match embryo viability. However, much of the egg laying curve parallels the hatching curve but does not overlap it (Figure 2A). This suggests a latency in the system that allows egg laying to continue for ∼1.5**°**C beyond embryonic viability. We hypothesized that this temperature difference may reflect a cost of halting egg laying in response to fluctuating temperatures. In an environment where the temperature changes frequently, it may be better to produce a few embryos that will not hatch rather than to completely stop laying eggs. We describe the test of this hypothesis below.

### Recovery of fecundity following chronic heat stress

Since we saw that embryo hatching declined during chronic heat stress, we wondered how other life history traits were affected by temperature. Much is known about the interaction of stress and adult life span [32]–[34] as well as trade-offs between reproductive capacity and life span [35]–[38]. To glean additional insights into lifespan and reproductive performance under stress, we measured adult viability and the ability to recover fecundity following stress over the same range of temperatures as egg laying and hatching (Figure 2B). Nearly all worms survived a 24-hour exposure to temperatures of up to 31**°**C, suggesting that *C. elegans* embryos cannot endure conditions beyond the tolerance of adult worms, but adult viability declined sharply at higher temperatures. The ability of adults to recover fecundity following a 24-hour heat stress initially declined in parallel with the decline in embryos’ ability to hatch. However, at 31**°**C there was a striking increase in the ability to recover fecundity following stress. This phenomenon was confirmed by examining recovery at additional temperatures above and below 31**°**C (Figure S1) and was also evident for heat stresses shorter than 24 hours (Figure S2, Figure S3). Whatever the cause of this peak, it serves to extend the range of temperatures that *C. elegans* can endure and still produce progeny.

### Chronic heat stress at 29°C is more damaging than at 31°C

**Figure 3:**
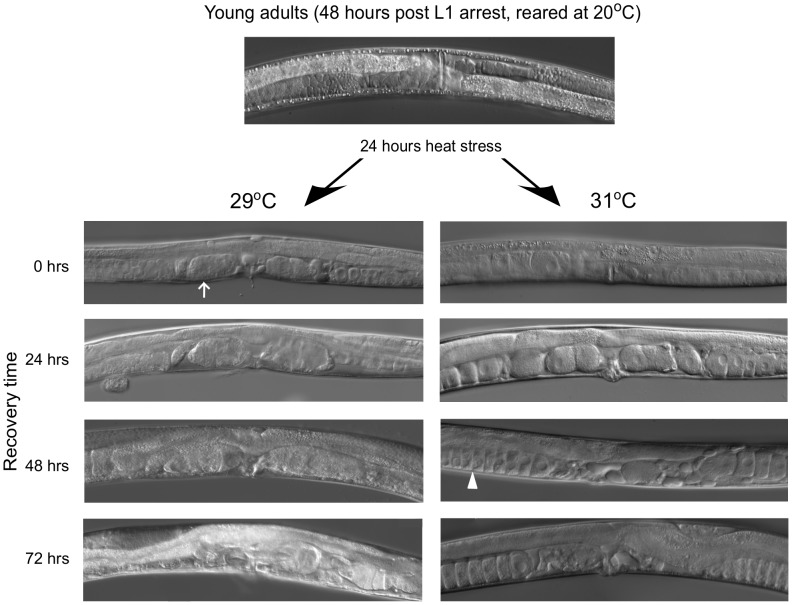
Reproduction-related defects following chronic heat stress. Representative images of worms at 0, 24, 48 and 72 hours of recovery from a 24-hour stress either at 29°C or 31°C are shown. An arrow points to uterine concretions and an arrowhead to stacked oocytes. In all pictures, anterior is to the left and ventral is down.

Surprised by the substantial increase in the ability to recover fecundity following a 31**°**C heat stress, we sought to identify the causes of this phenomenon. To visualize damage from stress, we compared individual worms following a 24-hour exposure to 29**°**C or 31**°**C (Figure 3). There was a noticeable difference in the appearance of these two groups of animals (Table 1). Worms treated at 29**°**C exhibited pronounced “concretions” of fertilized and unfertilized oocytes in the uterus (Figure 3, arrow in 29**°**C, 0 hours). These concretions resembled previously identified “oocyte clusters” [39] seen in aging hermaphrodites at the end of their reproductive life span. In addition, oocytes in the proximal gonads of these worms appeared shrunken and disorganized. After 24 hours of recovery, worms stressed at 29**°**C displayed even larger concretions and significant injury to the proximal gonad. In some, there were many small, irregular cells in the proximal gonad, a feature of poor quality oocytes [39], [40]. By 72 hours of recovery, some worms no longer had these concretions and may have been able to expel them from the uterus. Others showed internal hatching of embryos that would have eventually caused the hermaphrodite to “bag” [41].

**Table 1:** Uterine concretions and stacked oocytes in the proximal gonads of worms heat stressed at 29**°**C and 31**°**C.

Phenotype	Hours	29°C	31°C
Uterine concretions	0	0.98	0
	24	0.98	0.02
	48	0.82	0.02
	72	0.72	0
Stacked oocytes	0	0	0
	24	0	0.04
	48	0.24	0.66
	72	0.32	0.90

In contrast, the worms that had been stressed at 31°C (for 24 hours as above) and examined immediately afterward did not have concretions, and the oocytes in the proximal gonad were regular in size. After 24 hours of recovery, some of these worms had small concretions in the uterus including unfertilized oocytes. There was a rounding of the oocytes in the proximal gonad that produced small gaps between the cells. After 48 hours of recovery, worms displayed stacked oocytes (arrowhead in 31°C 48 hours) in one or both gonadal arms. Stacked oocytes are characteristic of a gonad that is producing oocytes but no longer has functional sperm to induce oocyte maturation and ovulation [40], [42], [43]. After 72 hours of recovery, almost all of the worms treated at 31°C had stacked oocytes in both gonads.

We interpreted these results to mean that worms exposed to lower stress temperatures (e.g. 29°C) experienced relatively greater damage to the gonad and the ability to produce oocytes than the worms exposed to 31°C. The latter animals were primarily affected by sperm loss. We next tested these hypotheses.

### Sperm defects are a major cause of fecundity loss after heat stress

Hermaphrodites produce a finite cache of sperm before irreversibly switching to oocyte production [44] and continue to produce oocytes until the end of their reproductive life span. Therefore, *de novo* production could replace oocytes, but not sperm, damaged by heat stress. Because mating during recovery provides unstressed sperm, it could be used to estimate the extent of damage that male gametes suffered during chronic stress. The “portion” of the wild type fecundity that could not be rescued by mating, provides an estimate of heat damage to the other components necessary for progeny production (e.g. oocytes and somatic gonad).

**Figure 4:**
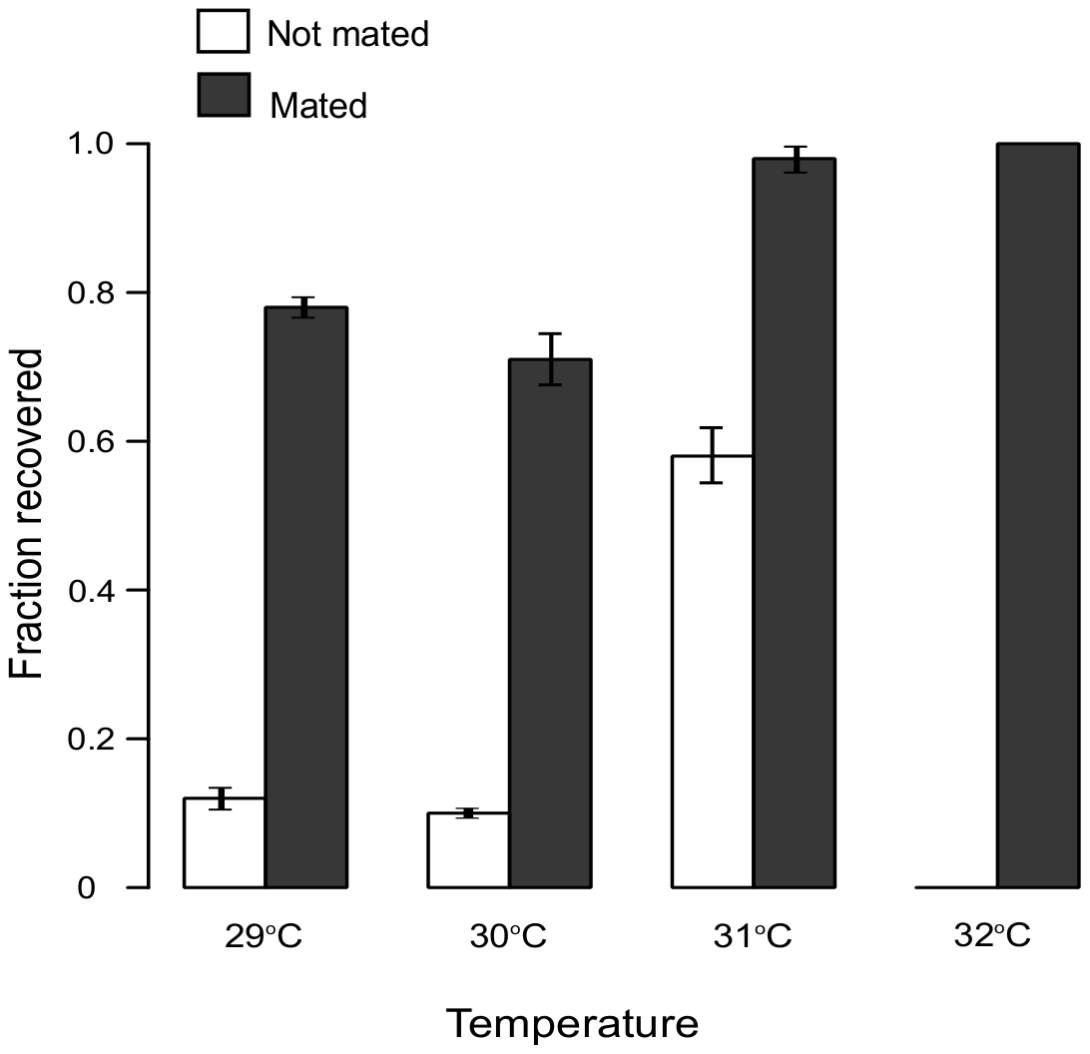
Mating greatly improves recovery of fecundity. The addition of unstressed males to hermaphrodites recovering from a 24-hour chronic heat stress greatly improves the fraction of worms that can produce offspring. Differences in recovery of fecundity between mated and not mated worms were significant for all comparisons. Binomial probabilities of observing as many or more recovered worms following mating, given the fraction that recovered without mating: 29°C (*p* = 1.4×10^−17^), 30°C (*p* = 3.6×10^−22^), 31°C (*p* = 2.4×10^−10^), 32°C (*p*<4.2×10^−85^). Error bars represent s.d.

We subjected hermaphrodites to 24 hours of stress at 29°, 30°, 31°, and 32°C (using the same protocol as above) but added unstressed males during the recovery period. As expected, this increased the fraction of worms that recovered fecundity (Figure 4) even for worms exposed to 32°C, which could not recover on their own. Because nearly all worms treated at 31°C or 32°C produced offspring when mated, we concluded that at these temperatures oocytes and/or the somatic gonad could survive the stress sufficiently intact to allow at least some recovery. Lower recovery of worms exposed to 29°C and 30°C suggested irreparable damage to these components of the reproductive system.

### Heat stress damages oocyte production

**Figure 5:**
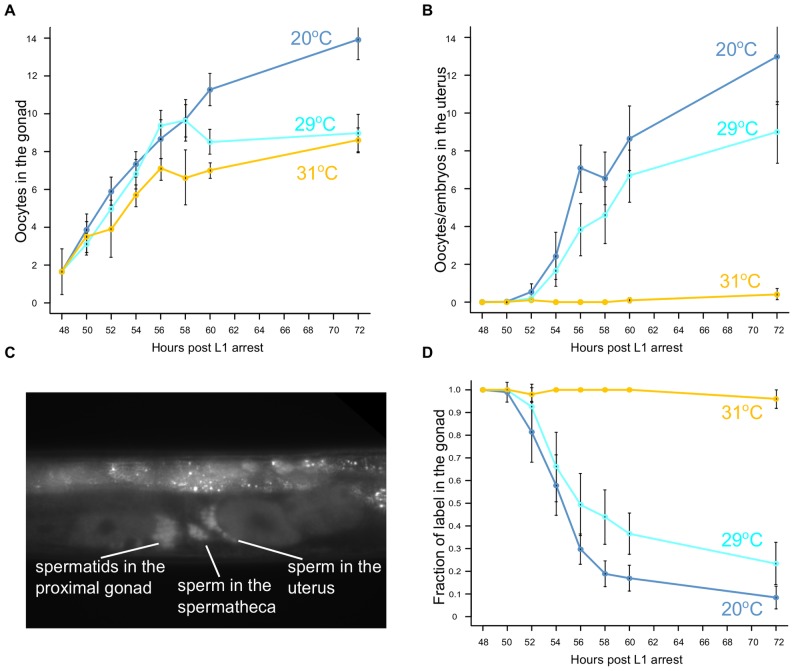
Oocyte production, ovulation, and spermatid transit at different stress temperatures. (A) Oocyte production at stress temperatures (29°C and 31°C) compared to the 20°C control. (B) Ovulation at stress temperatures (29°C and 31°C) compared to the 20°C control. (C) A representative image of mCherry-expressing spermatids and sperm in a young adult (54 hours post L1 arrest) hermaphrodite, showing the partitioning of labeled cells between the proximal gonad and the spermatheca and uterus. (D) Transit of spermatids from proximal gonad at stress temperatures (29°C and 31°C) compared to the 20°C control. Error bars represent s.d.

We examined the effects of heat stress on three subsystems of the reproductive system: oocyte production, ability to ovulate, and sperm production. In *C. elegans,* hermaphrodites reared at 20°C are self-fertile and make approximately 300 spermatids during the L4 larval stage. They irreversibly switch to producing oocytes after the molt that defines the transition to adulthood [45]–[47] and continue to make oocytes until reproductive senescence [38], [39]. Spermatids remain in the proximal gonad until ovulation of mature oocytes push them into the spermatheca where they are activated to produce mature sperm [44], [48]. We examined synchronized young adult worms every two hours for the production of oocytes, ovulation, and spermatid activation. First we counted oocytes in the proximal portions of both the anterior and posterior gonadal arms (Figure 5A, Figures S4–S7). We show the total results for both gonadal arms, but consistent with the observations of Ward and Carrel [44] on spermatid maturation, oocyte production in the anterior gonad preceded production in the posterior gonad by ∼1 hour (Figure S4). At 48 hours post L1 arrest, young adults reared at 20°C had on average two oocytes in the proximal gonad, indicating that the switch from spermatogenesis to oogenesis had occurred. At 29°C, oocyte production did not appear markedly different until ten hours of heat stress, when production slowed. Oocyte production was slower yet at 31°C, but arrest of egg laying at 31°C clearly cannot be due to failure to produce oocytes.

### Heat stress effects on ovulation and spermatid activation

Along with oocyte production, we counted embryos in the uterus. At temperatures below 28°C, all of these would be fertilized embryos; at higher temperatures, it was often difficult to distinguish between the fertilized and unfertilized eggs. Importantly, at 31°C, ovulation never commenced (Figure 5B). Young adult worms were shifted to the heat stress temperature after 48 hours of development (starting at L1 arrest). Because ovulation begins at ∼51 hours at 20°C, we concluded it took less than three hours of heat stress at 31°C to prevent ovulation.

In *C. elegans* hermaphrodites, spermatids produced during the L4 stage remain in the proximal gonad until the ovulation of the most proximal oocyte pushes them into the spermatheca where they are activated to become sperm capable of fertilization [44], [49], [50]. In this way, spermatid activation is dependent on ovulation. While it has been noted that not all of the spermatids are activated with the first ovulation (some remain in the proximal gonad after the first ovulation [44], [51]), the dynamics of this process have not been quantified. We used a strain in which spermatids and sperm were expressing mCherry [52] to follow the transit of labeled spermatids from the proximal gonad to the spermatheca and uterus (sperm are carried into the uterus by ovulation, but are able to crawl back into the spermatheca [44]). A synchronous population of worms was examined for the presence of the mCherry label in each of the three compartments–proximal gonad, spermatheca, and uterus (Figure 5C). We took the transit from the proximal gonad as a proxy for spermatid activation, since the sperm that have not moved to the spermatheca or uterus remain as spermatids. The fraction of label remaining in the proximal gonad of the anterior gonad arm is shown in Figure 5D. The dynamics of transit in the posterior gonad arm were similar but lagged by about an hour (Figure S8, Figure S9).

At 20°C, spermatid transit was neither instantaneous nor complete (Figure 5D). Approximately 50% of the mCherry label moved from the proximal gonad during the first six hours. During this time ∼2 oocytes were ovulated from the anterior gonad. Some spermatids were present in the proximal gonad after twelve hours, a time that corresponds to about 16 ovulations from the anterior arm (∼4 embryos from the anterior arm stored in the uterus (Figure 5B) and 12 embryos from the anterior arm laid [21]). Thus even multiple ovulations were not sufficient to expel all spermatids into the spermatheca. We saw small numbers of labeled spermatids remaining in the proximal gonad in worms as old as 100 hours post L4/adult molt (data not shown). Ovulation and spermatid transit were only slightly less efficient at 29°C (Figure S10) and 30°C (Figure S11). In sharp contrast, at 31°C, ovulation occurred only rarely and spermatids remained in the proximal gonad (Figure 5D, Figure S12) and were not activated to form mature sperm.

### Mutants with altered ovulation dynamics recover differently

**Figure 6:**
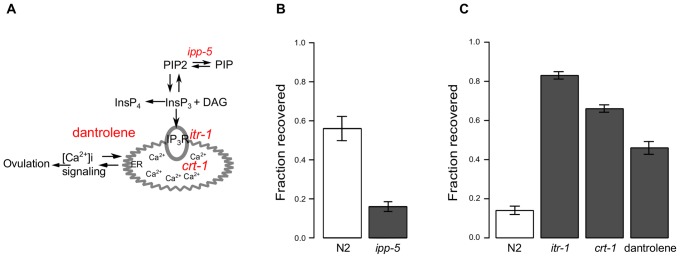
Mutants with altered ovulation dynamics recover from heat stress differently than the wild type. (A) Schematic depiction of several key components of Ca^2+^ signaling that regulates ovulation. Modified from [53]. (B) Recovery of fecundity (after a 24-hour heat stress at 31°C) in a mutant with up-regulated Ca^2+^ signaling is lower than in a wild type control (binomial test *p* = 4.3×10^−9^). (C) Recovery of fecundity (after a 24-hour heat stress at 29°C) of mutants or chemical treatment that down-regulate Ca^2+^ signaling is higher than in a wild type control: *itr-1* (binomial test *p* = 7.7×10^−28^), *crt-1* (*p* = 2×10^−25^), and dantrolene (*p* = 1.6×10^−8^). Error bars are s.d.

To test the idea that continued ovulation at high temperatures leads to increased damage and decreased recovery, we examined mutants with altered rates of ovulation. In *C. elegans*, the inositol 1,4,5-trisphosphate Ca^2+^ signaling pathway controls rhythmic behaviors including pharyngeal pumping, defecation, and ovulation [53]–[57]. We considered a number of mutants in this pathway to examine the recovery of worms with altered ovulation dynamics (Table S3) and tested those mutants that were amenable to our protocol (genes are highlighted in red, Figure 6A). We expected that worms that had greater Ca^2+^ signaling would have increased rates of ovulation at 31°C and incur greater damage to both sperm and somatic gonad. These worms would therefore be less likely to recover fecundity. One such mutant, *ipp-5(sy605)*, a loss-of-function allele of the type 1 inositol polyphosphate 5-phosphatase, ovulates more than one oocyte per cycle [54]. Unlike wild type N2, most worms of this strain continued to ovulate a few oocytes at 31°C (Figure S13). As predicted, a much smaller fraction of these mutants were able to recover fecundity after being subjected to heat stress at 31°C (Figure 6B).

Conversely, we hypothesized that worms that had decreased Ca^2+^ signaling would have decreased rates of ovulation at 29°C and would recover better than N2. We selected two such mutants. The first, a gain-of-function allele of *itr-1(sy327)* [55] has increased Ca^2+^ signaling, but also increased length of ovulatory contractions [57], meaning that the effective rate of ovulation for this mutant is decreased (Figure S14). A much greater fraction of these worms recovered from a 29°C stress compared to wild type (Figure 6C). We also considered a loss-of-function mutant in this same gene, *itr-1(sa73)* [55], but these worms have aberrant ovulation [54], [57] and did not recover fecundity after heat stress (results not shown). The second mutant was an allele of calreticulin, a calcium binding protein found in the endoplasmic reticulum which functions as a chaperone under heat stress [58]. The *crt-1(jh101)* mutant has both decreased fertility at 25°C and defective sperm. Nonetheless, *crt-1(jh101)* has a decreased rate of ovulation (Figure S14) and recovered fecundity better than wild type after heat stress at 29°C (Figure 6C). We examined these mutants during the course of recovery but did not observe overt differences compared to the wild type (data not shown).

In addition to mutations that decrease ovulation, we tested dantrolene, a substance that inhibits release of calcium from the endoplasmic reticulum [59]. We expected treated worms to have decreased Ca^2+^ signaling and thus decreased ovulation rates. Slower ovulation would cause them to sustain less damage to the somatic gonad. We treated N2 worms with dantrolene sodium and saw that it effectively decreased the rate of ovulation (Figure S14). Importantly, a greater fraction of worms treated with this drug recovered fecundity than untreated worms (Figure 6C).

Altogether these data are consistent with our hypothesis that ovulation at high temperature leads to greater damage to the somatic gonad and inhibits recovery of fecundity after heat stress. In contrast, worms that perform fewer ovulations incur less damage to both mature sperm and the somatic gonad and are more likely to produce progeny after heat stress.

### Trade-offs in the recovery from heat stress

Worms that were heat-stressed at 29°C continued to ovulate and incurred more damage than worms that were heat-stressed at 31°C and never began to ovulate. Since ovulation is obviously detrimental under prolonged heat stress, why don’t worms stop ovulating at the damaging 29°C? One possible explanation is that the benefit of stopping ovulation is counterbalanced by a cost. A plausible candidate for such a cost is the length of time it may take to restart reproduction following cessation of ovulation. Worms that continue ovulating through somewhat adverse conditions might recover faster and outcompete the worms that stop. Hodgkin and Barnes [60] developed a protocol to compare rates of population growth among worm strains, the “eating race”, which we used to test this hypothesis.

**Figure 7:**
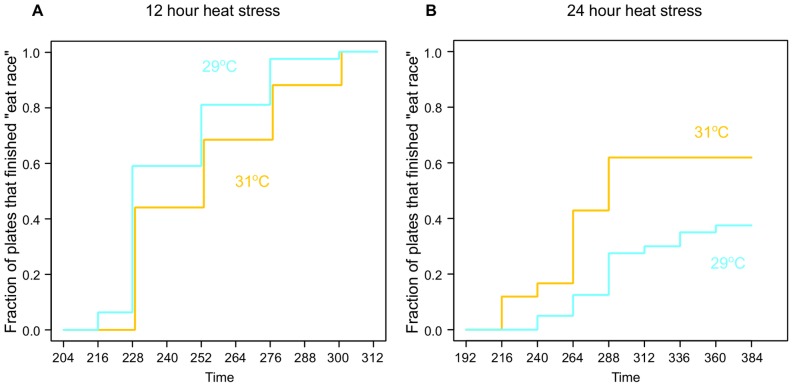
Duration of heat stress determines whether stopping or continuing reproduction is the advantageous strategy. Curves represent the fraction of plates (each containing offspring of a single stressed individual) that have exhausted bacterial food and thus finished the “eating race”. Because all plates initially contained equal amounts of bacteria, plates that were consumed faster contained more offspring. Depending on the duration of the stress, (A) 12 and (B) 24 hours, winning strategies were continued reproduction (worms stressed at 29°C continued to lay eggs) or cessation (worms stressed at 31°C did not ovulate or lay eggs). Time is in hours. The difference between the two curves was suggestive, although not significant in panel A (Kolmogorov-Smirnov test *p* = 0.13) and significant in panel B (*p* = 1.7×10^−4^).

We reasoned that if the duration of chronic stress is relatively short, the cost of continuing to lay eggs (greater damage to the reproductive system) might be compensated by faster recovery after the stress is over. We tested this notion by conducting eating races between worms treated for 12 hours at 29°C and those treated for 12 hours at 31°C. Indeed, worms subjected to heat stress at the lower temperature finished the eating race faster (Figure 7A). Even though both sets of worms eventually recovered at the same frequency and with similar average brood sizes, worms that had not stopped ovulating were able to produce the next generation of offspring sooner.

On the other hand, when this experiment was conducted for 24 hours of heat stress, worms exposed to higher temperature recovered faster (Figure 7B). We interpreted this as evidence that at this duration of stress, the amount of damage incurred by the reproductive system while ovulating at temperature was too great to be overcome by a quicker recovery of egg laying.

## Discussion

We investigated reproductive performance of *C. elegans* under chronic heat stress. We found that worms could tolerate rather prolonged exposure to relatively high temperatures and still retain the ability to reproduce. Perhaps not surprisingly, longer stress durations and higher temperatures generally cause greater damage and reduce the likelihood of recovery. A prominent exception to this principle was observed when worms were subjected to stress at 31°C and performed better than worms that had been stressed at lower temperatures. We interpret the dramatic departure from the “expected” relationship between stress and performance (Figure 2B) as evidence for two distinct strategies for coping with stress (more on this below).

Examination of worms following stress revealed that both oocytes and sperm suffer from elevated temperatures. However, because only a fixed cache of the latter is produced, whereas oocytes are generated continuously until reproductive senescence [61], the number of surviving sperm limits the extent of self-recovery. This is potently demonstrated by the substantially improved recovery upon mating with unstressed males (Figure 4).

Two lines of evidence suggest that whether worms initiate ovulation is a regulated “decision” based on the assessment of the environment. First, there is a sharp difference between the rates of ovulation at 30°C and 31°C (Figure 5, Figures S4–S8, Figures S10–S12). Second, *ipp-5* mutants continue ovulation at 31°C (Figure 6, Figure S13), suggesting that this temperature does not represent an absolute physical limit to ovulation. For this reason, we favor a hypothesis that ovulation is a key determinant of recovery, although we could not completely rule out a contribution of systemic heat stress response.

We think that the peculiar shape of the recovery curve (Figure 2B) reflects the fact that it consists of several distinct components. Self-recovery, that is, the ability to produce self-offspring, obviously depends on the presence of viable oocytes and sperm. The increasing part of the peak centered on 31°C is due to the fact that at higher temperatures fewer animals initiate ovulation therefore mitigating damage to the gonad and gametes. The decreasing part of the peak corresponds to spermatid death. In this way, 31°C can be seen as a temperature at which these two curves happen to intersect. In other words, the increasing rate of spermatid death at higher temperatures limits the probability of recovery.

Understanding that there is considerable benefit to not ovulating at heat stress temperatures, we wondered why worms continue to ovulate under the obviously damaging conditions of 29°C. The “eating races” following heat stress (Figure 7) demonstrate that the duration of stress determines which of the two “strategies” (stop vs. continue) is advantageous. After a short (12 hours) stress, continued egg laying resulted in a faster population recovery, whereas after a long (24 hours) stress, cessation of egg laying yielded better results. Of course when *C. elegans* encounter high temperatures, the worms cannot predict how long the stress will continue. Our results suggest that the worms may interpret stress temperature as a proxy for stress duration. It has been suggested previously that because some environmental parameters are correlated in natural habitats, organisms may act in “anticipatory” fashion towards one variable, by simply responding to another [62], [63]. If true, worms may be relying on an easy-to-detect [64] variable (stress temperature) to make judgments about an inherently unpredictable variable (stress duration). The specific settings of the multi-component IP3 signaling system, which regulates ovulation [65]–[70], may reflect an adaptation to life under the particular variable environmental conditions in which *C. elegans* evolved.

At first glance it may be puzzling why worms continue egg laying at temperatures at which none of their offspring could survive (Figure 2A). Thinking about continued ovulation at 29°C as a trade-off for a potential rapid recovery, offers a straightforward explanation. If stress only lasts a short time, the cost of losing offspring unable to hatch during stress would be compensated by rapid reproductive recovery. On the other hand, worms that stop reproduction, better preserve their reproductive ability, but take longer to re-initiate egg laying.

To optimize long-term growth rates in fluctuating environments, organisms adopt reproductive strategies that allocate some gametes for active offspring production, while saving up others; the ratio depends on the past and present environmental conditions [6]. Not only plants and animals, but also bacteria [3], [4] and yeast [71] engage in trade-offs between maximizing reproductive rates under favorable conditions and survival in detrimental environments. A previous study using *C. elegans* reported a trade-off between stress resistance and reproductive fitness [72]. Here we described evidence of a trade-off between two alternative strategies for dealing with heat stress, namely continuing egg laying vs. reproductive cessation. We expect this work to illuminate the mechanism that allows *C. elegans* to survive in unpredictably variable environments.

## Materials and Methods

The phenotypes observed in these experiments are robust and reproducible. Great care was taken to standardize experimental procedures (see KEY ELEMENTS OF EXPERIMENTAL DESIGN below). Using these procedures naïve undergraduate students have been able to contribute to our data sets. Without systematic quality controls, even the most experienced researcher will be frustrated by inconsistent phenotypes.

### Preparation of Worm Plates

60 mm non-vented petri dishes were filled with 10 mL of NGM medium [73]. Plates were allowed to age for one day before use. Excess moisture was removed by shaking droplets off plates. We did not dry plates in the incubator – this causes a separation between the edge of the plate and the medium and worms will crawl into the void and disappear. Plates were dried further when seeded.

We streaked new plates of OP50 on LB agar monthly and stored them at 4°C until needed. Fresh cultures of OP50 were made by inoculating a single colony from a streak plate of OP50 into 10 mL of LB broth. Liquid cultures were incubated at 37°C overnight with shaking. Lawn plates were made by seeding 60 mm plates of NGM agar with 100 uL of the overnight culture and spreading the culture with a sterile bent glass rod and allowed to grow at room temperature overnight. We seeded each plate for singled worms with a 5 uL drop of a 1∶1000 dilution of the overnight culture of OP50. We took care not to puncture the surface of the agar when seeding since this encourages the worms to burrow. The small bacterial spot allowed all the worms to be observed under the dissection scope at one time. All plates were seeded with lids ajar within 12 inches of an open Bunsen burner to avoid contamination and facilitate the loss of excess moisture.

### Temperature Consistency

Consistency in temperature is critical to the reproducibility of these experiments. Since our heat shift experiments were initially performed in conjunction with a laboratory at another university, we took extra care to calibrate our incubators. We used large Percival incubators for our experiments. There is an excellent discussion of spatial variability within incubators in the Methods section of Ailion and Thomas [74] and we generally followed their methods. Four VWR digital recording thermometers with accuracy to 0.1°C were checked against each other and used to calibrate each of four incubators that were kept at the following temperatures: 20°C, 25°C, 29°C and 31°C. After this initial calibration period, one recording thermometer was kept in each incubator. These incubators were occasionally adjusted to other temperatures as required. At the beginning of each temperature shift experiment, the recording thermometer was set and results from experiments in which the temperature fluctuated more than 1.0°C were excluded. We used 50 plates of singled worms for each replicate. The plates were rubber-banded together in stacks of five (agar side up) and placed in a covered plastic shoebox. Boxes of plates were incubated in the middle of the incubator next to the thermometer probe. We looked for and did not observe any systematic differences in recovery due to the position of individual plates.

### Worm Strains and Maintenance

*C. elegans* strains were maintained at 20°C using standard methods as described previously [75]. The following strains were used: wild type N2 Bristol, EG4883 *oxIs318[pCFJ167(Pspe-11::mCherry::histone – Cbr-unc-119(+))]* II *unc-119(ed3)* II, JT73 *(itr-1(sa73)* IV, KJ216 *crt-1(jh101)* V, PS2368 *itr-1(sy327) unc-24(e138)* IV, PS3653 *ipp-5(sy605)* X.

Synchronized cultures of L1 larvae were prepared by hypochlorite treatment of gravid hermaphrodites [76]. Care was taken to use populations of worms that were well fed and did not contain dauers, or males. The effects of undergoing transition to dauer persist in the population [77], while males are a hallmark of populations that have experienced stress [78]. If dauer worms or males were detected in the population, a fresh population was started using L4 hermaphrodites and was propagated for three generations before use. The liberated eggs were allowed to hatch in M9 buffer overnight and the arrested L1 larvae were plated the next morning.

We used a micropipette to transfer 5 uL drops from the egg preparation to the lawn plates. The time that L1 worms were plated was noted as “0 hours post L1 arrest” and ages were measured from this time point. We transferred between 30 and 50 L1 larvae to each lawn plate. It is important to keep the density of worms consistent since developmental times are dependent on the density of worms. If several 5 uL drops were required, we spaced the drops evenly on the plate to ensure uniform absorption of the M9 buffer into the plates. The L1 larvae were plated onto NGM agar plates with a confluent lawn of *E. coli* OP50. This assured that the L1 larvae would start feeding wherever they landed and improved synchronization (confirmed by microscopic examination of worms at 44 hours post L1 arrest and was greater than 92%).

For some experiments, synchronized cultures of L1 larvae were prepared using a one-hour timed egg lay. For these experiments, worms were synchronized using the hypochlorite treatment described above. L1s were plated as described above and incubated for 72 hours. Hermaphrodites from these populations were used for one-hour egg lays. We saw no difference in results depending on which method was used for synchronization.

### Staging Worms

To standardize the developmental stage when young adults were shifted to heat stress temperature, worms were first singled just after the L4/adult molt (46 or 47 hours after plating). Just after singling, several worms left on the synchronized population plates were examined using a compound microscope. We counted the number of oocytes in both arms of the proximal gonad. When the population of worms had an average of one oocyte per gonad (generally at 48 hours post plating of L1s) we concluded that the switch from spermatogenesis to oogenesis had occurred and the singled worms were shifted to the heat stress temperature. Mutants in the inositol 1, 4, 5-trisphosphate Ca^2+^ signaling pathway were staged using the same protocol. These data are shown in Table S4.

### Heat Stress and Recovery

At t = 0, (48 hours post L1 arrest) 50 plates each containing a single adult hermaphrodite were shifted to temperature. After the designated period of heat stress, the worms were returned to 20°C and the minimum and maximum temperatures over the course of the temperature shift were noted. Over the next 120 hours, worms were checked daily and the numbers of eggs, both fertilized and unfertilized, and larvae were recorded. The results from worms that didn’t survive to the end of the experiment were not included. Animals were scored as dead if they were unresponsive to touch and did not exhibit pharyngeal pumping. A schedule for an experiment measuring the recovery of fecundity after a 24-hour heat stress is provided in Table S5.

### Mating Rescue

Mating rescue experiments were performed as the recovery experiments described above except that after heat stress, half of the heat stressed worms were mated to fresh males. Three 72-hour post-hatch N2 males, which were reared at 20°C, were added to each plate.

### Oocyte Census and Sperm Transit

Oocyte census experiments were performed using synchronous cultures of both N2 and EG4883. The numbers were comparable between the two strains. Every two hours, beginning at 48 hours post L1 arrest, twenty-five worms were examined under a compound microscope. Only some oocytes were large enough to fill the lumen of the gonad entirely. We counted these oocytes and the number of fertilized embryos in the uterus, in both the anterior and posterior arms. In addition, we determined the average age at which hermaphrodites reared at 20°C laid their first egg. To do so, individual hermaphrodites were singled from synchronized populations of worms and monitored every 30 minutes.

Sperm transit experiments were performed using synchronous cultures of EG4883. This strain carries an mCherry-histone fusion under the control of the *spe-11* promoter, which labels both spermatids in the gonad and activated sperm in the spermatheca and uterus. Every two hours, beginning at 48 hours post L1 arrest, worms were immobilized in 100 mM sodium azide and examined on a compound microscope. The strength of the mCherry signal was assigned a value between 1 and 10, and the compartment in which it was present– proximal gonad, spermatheca, and uterus – was noted. The value of 10 indicated that all label was in the proximal gonad, while the score of 1 was given for the lowest amount of label retained there.

### Microscopy

Worms were examined on a Leica DM5000B compound microscope. Images were taken with a Retiga 2000R camera and analyzed using ImageJ software. Individual images were stitched together using the MosaicJ plug-in [79].

### Egg Hatching

Ten gravid 72-hour post hatch adults were allowed to lay eggs for one hour at room temperature. The adults were removed and the plates were immediately incubated at heat stress temperature for 24 hours, at which time the numbers of larvae and unhatched eggs were counted. The plates were incubated at 20°C for an additional 24 hours and reexamined to make sure embryos did not arrest in the heat.

### Recovery on Dantrolene

A synchronized culture of N2 was prepared by hypochlorite treatment of hermaphrodites as above. The culture was plated on lawn plates of OP50 on NGM supplemented with 10 uM dantrolene. The worms were raised on these plates and shifted to 29°C. After 24 hours at temperature, the plates were shifted to 20°C and single worms were transferred to individual OP50-seeded NGM plates without dantrolene and allowed to recover.

### Eating Races

Fifty 48-hour post L1 arrest worms from the same synchronized culture were singled on plates and heat stressed as described above. Twenty-five of these plates were shifted to 29°C and, at the same time, twenty-five plates were shifted to 31°C. Worms were at heat stress temperature for 12 or 24 hours and then returned to 20**°**C. Each worm was allowed to recover on a plate without bacteria for 20 minutes and then singled onto new seeded test plates with carefully matched sizes of OP50 bacterial lawns. Plates were monitored daily for the consumption of the bacteria and the production of larvae. Worms “finished” the race when all bacteria were consumed.

### Key Elements of Experimental Design

Although our experimental designs are straightforward, considerable effort has gone into their optimization. Our experience led us to conclude that even minor deviations from protocol can substantially increase the variability of many phenotypes. With that in mind, we outline some procedures to increase experimental reproducibility. Earlier, we detailed efforts to standardize the media, food quality and amount, temperature consistency, and the quality, developmental synchronicity, and density of the worm populations. Here we discuss more generally why stringency is the essential component to all of the protocols we developed.

Central to our experimental design is the notion that “*a* worm is not *the* worm”. Despite being effectively isogenic, *C. elegans* individuals display a remarkable amount of phenotypic variation [80]–[82]. By examining large numbers of worms, we can discern and evaluate the variance of phenotypes that might have been missed otherwise. For example, previously it was suggested that a 30**°**C heat stress is sufficient to cause complete sterility [17]. By examining large numbers of individuals we were able to show that some worms can recover fecundity after such treatment (Figure 2B). Large sample sizes also help to reduce the effect of experimenter-induced variance (Table S2, Table S6).

“Young adult”, “pregravid” and even “prefertile” are all terms that have been used to describe adult worms before they begin laying eggs, although unlike “L1 larva” or “dauer larva”, they have no standard definition in the lexicon of *C. elegans* development, and the time period they refer to encompasses a number of events that are important for the development of sexual maturity. The time between the molt that produces the adult stage of *C. elegans* and the onset of egg laying is about nine hours at 20**°**C [20], [21]. During that time period, while continuing to grow, hermaphrodite worms cease producing sperm, begin producing oocytes, begin ovulation, spermatids mature and become sperm, fertilization commences, and the first egg is laid [44]. Our experiments therefore required a more precise staging, which is why we measured worm development in “hours post L1 arrest at 20**°**C” [83] and mapped some of the above developmental landmarks against time (Figure S15) with the caveat that the absolute timing of different laboratory strains may vary.

We systematically examined hundreds of individual worms to determine the median time (at 20**°**C) for the appearance of the first oocyte (47.5 hours post L1 arrest), the first ovulation (52.5 hours post L1 arrest) and the first egg laid (54.5 hours post L1 arrest) (Figure S15). We found that the anterior arm of the gonad produces the first oocyte somewhat earlier than the posterior arm. We conducted temperature shifts at 48 hours post L1 arrest because by this time, at 20**°**C, almost all hermaphrodites had one oocyte in the anterior gonad (demonstrating that the switch to oogenesis had taken place) and most had one oocyte in the posterior gonad, but no worms had begun ovulation.

The rate of development changes with temperature [20] and high temperature produces an alternate form of development – the dauer larvae [74]. These facts were considered when choosing the time point to perform temperature shift experiments. First, because we did not want any temperature-dependent aspects of larval development to confound interpretation of our experiments, we chose to apply temperature stress at a developmental stage that would not interfere with production of adult hermaphrodites. Second, hermaphrodites produce sperm during the last larval stage and switch to oocyte production after the larval to adult molt [45], [47], [84]. The presence of oocytes could therefore provide a landmark for the stage of maturity at which to perform the temperature shift (the absolute time differed between different temperatures of incubation).

Our previous work convinced us of the importance of collecting accurate time-resolved data in order to understand dynamic processes [21]. To determine the timescale over which to sample, it is necessary to understand the dynamics of the process being studied. For instance, while measuring spermatid transit, we saw substantial differences when sampled every two hours. On the other hand, since it takes about 12 hours for *C. elegans* embryos to hatch [85], we decided to count the production of larvae during recovery from heat stress just once per day. Likewise, it is critical to understand the phenotypic parameters involved. Temperature, in particular, is known to produce non-linear responses in development [74], [86], [87]. In this paper, we interrogated chronic heat stress, across temperatures from 28**°**C to 33**°**C, by degree. These were tedious experiments to be sure, but the findings presented here would not have come to light except for this rigorous examination of the phenotypic space.

## Supporting Information

Figure S1 **Recovery of fecundity following 24 hours of stress at temperatures around 31°C.** Experiments were performed as described in Figure 1E. Numbers of worms for each temperature: 30**°**C (100), 30.5**°**C (50), 31**°**C (100), 31.5**°**C (50), 32**°**C (50).
(PDF)

Figure S2 **Reproduction across a range of chronic stress temperatures (12 hours of heat stress).** Experiments were performed exactly as described in Figure 2, except the duration of the heat stress was 12, not 24 hours. Note that egg hatching is identical to the results for 24 hours heat stress (Figure 2), therefore the effects of heat stress on embryos must take place during the first 12 hours.
(PDF)

Figure S3 **Reproduction across a range of chronic stress temperatures (18 hours of heat stress).** Experiments were performed exactly as described in Figure 2, except the duration of the heat stress was 18, not 24 hours.
(PDF)

Figure S4 **Census of oocytes in the proximal gonad and embryos in the uterus for worms kept at 20°C.** Oocyte production in the anterior gonad arm precedes production in the posterior arm by about an hour. Error bars are s.d.
(PDF)

Figure S5 **Census of oocytes in the proximal gonad and embryos in the uterus for worms shifted to 29°C at 48 hours post L1 arrest.** Error bars are s.d.
(PDF)

Figure S6 **Census of oocytes in the proximal gonad and embryos in the uterus for worms shifted to 30°C at 48 hours post L1 arrest.** Error bars are s.d.
(PDF)

Figure S7 **Census of oocytes in the proximal gonad and embryos in the uterus for worms shifted to 31°C at 48 hours post L1 arrest.** Error bars are s.d.
(PDF)

Figure S8 **Spermatid transit at 20°C.** The distribution of the mCherry signal between the three compartments - proximal gonad, spermatheca, and uterus – of both the anterior and posterior gonad arms. Error bars are s.d.
(PDF)

Figure S9 **Schematic depiction of the reproductive system of** ***C. elegans*** **hermaphrodite after the first ovulation.** The first ovulation typically occurs in the anterior gonad (left). The passage of the oocyte pushes some spermatids into the spermatheca where they become mature sperm capable of amoeboid movement. The oocyte is fertilized in the spermatheca and passes to the uterus. The posterior gonad (right) is shown prior to the first ovulation. Immature spermatids are concentrated in the proximal gonad.
(PDF)

Figure S10 **Spermatid transit for worms shifted to 29°C at 48 hours post L1 arrest.** Error bars are s.d.
(PDF)

Figure S11 **Spermatid transit for worms shifted to 30°C at 48 hours post L1 arrest.** Error bars are s.d.
(PDF)

Figure S12 **Spermatid transit for worms shifted to 31°C at 48 hours post L1 arrest.** Error bars are s.d.
(PDF)

Figure S13 **Fraction of N2 and** ***ipp-5(sy605)*** **worms that ovulated at 31°C.**
(PDF)

Figure S14 **Ovulation during heat stress (29°C) in wild type and mutant worms.** The average number of embryos in the gonad (left) and eggs laid (right) for N2, *itr-1*(*gf*), *crt-1* and N2 raised on 10 uM dantrolene. The total number of ovulations at 29**°**C can be obtained by adding values in each of these two categories.
(PDF)

Figure S15 **Timing of three milestones in the development of young adult hermaphrodites at 20°C.** Fraction of worms that have (A) produced the first oocyte, (B) begun to ovulate and produced the first embryo in the uterus. At each time point, multiple worms were observed. Fractions, not timing of events in individual animals are shown because once observed, animals could not be easily recovered. In contrast, (C) shows timing of first egg lay among 100 individually tracked worms.
(PDF)

Table S1 **Summary of results on recovery after heat stress.**
(PDF)

Table S2 **Primary data from heat stress and recovery experiments.**
(XLSX)

Table S3 **Mutants of the inositol 1,4,5-trisphosphate Ca^2+^ signaling pathway.**
(PDF)

Table S4 **Developmental staging of ovulation mutants.** The age at temperature shift reflects the degree that pleiotropic effects of mutations delayed development compared to wild type.
(PDF)

Table S5 **Timeline for a 24-hour heat stress experiment.**
(PDF)

Table S6 **Summary of the number of individual worms tested in all experiments.**
(XLSX)
