# Supplementary material for: Balanced Trade-Offs between Alternative Strategies Shape the Response of C. elegans Reproduction to Chronic Heat Stress
Source: PLoS One. 2014 Aug 28;9(8):e105513. doi: 10.1371/journal.pone.0105513 (PMC4148340; doi:10.1371/journal.pone.0105513)
Supplement: Figure S2 — Reproduction across a range of chronic stress temperatures (12 hours of heat stress). Experiments were performed exactly as described in Figure 2, except the duration of the heat stress was 12, not 24 hours. Note that egg hatching is identical to the results for 24 hours heat stress (Figure 2), therefore the effects of heat stress on embryos must take place during the first 12 hours. (PDF) [file pone.0105513.s002.pdf]

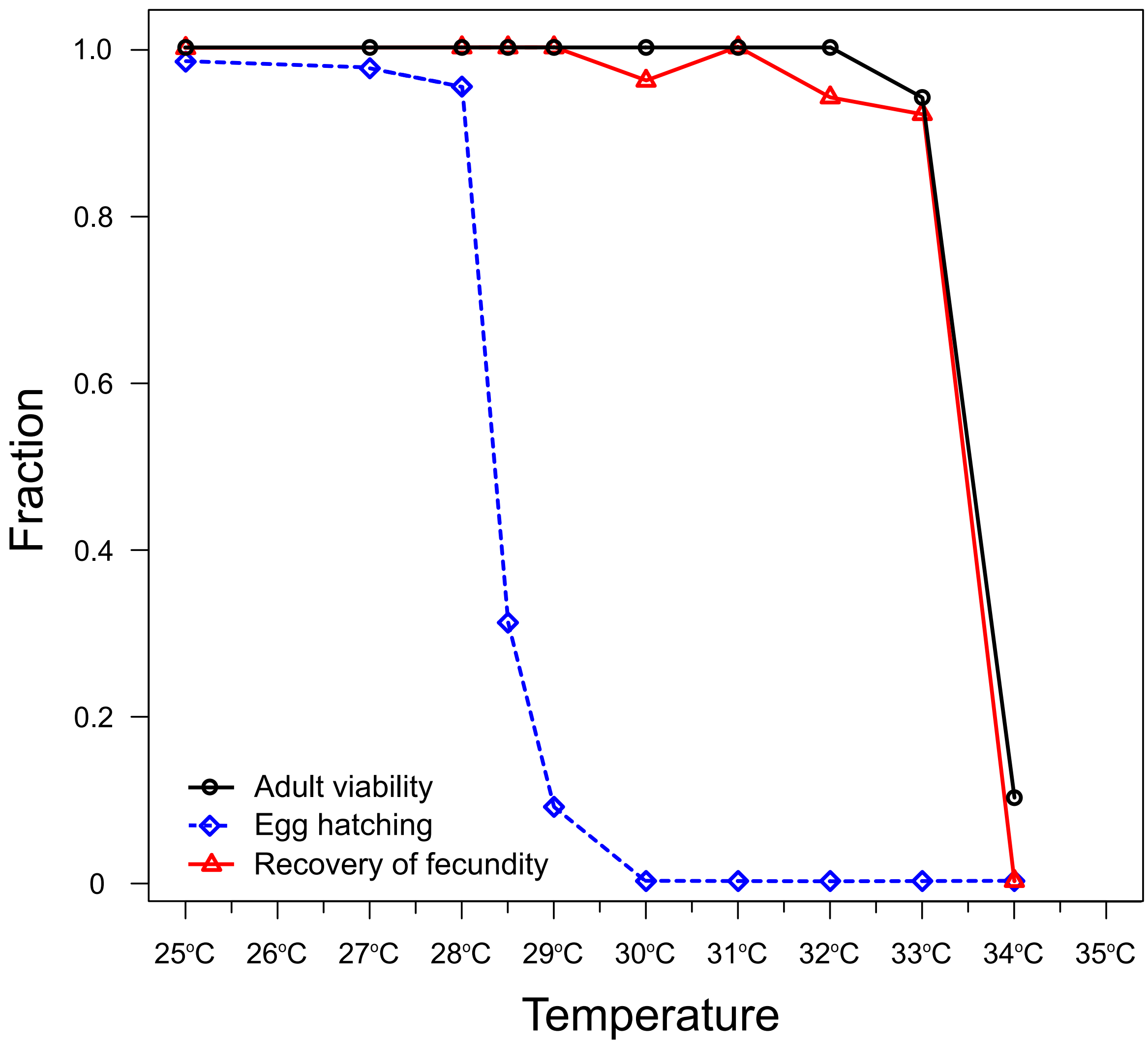

Figure S2. Reproduction across a range of chronic stress temperatures (12 hours of heat stress). Experiments were performed exactly as described in Figure 2, except the duration of the heat stress was 12, not 24 hours. Note that egg hatching is identical to the results for 24 hours heat stress (Figure 2), therefore the effects of heat stress on embryos must take place during the first 12 hours.
