# Supplementary material for: Balanced Trade-Offs between Alternative Strategies Shape the Response of C. elegans Reproduction to Chronic Heat Stress
Source: PLoS One. 2014 Aug 28;9(8):e105513. doi: 10.1371/journal.pone.0105513 (PMC4148340; doi:10.1371/journal.pone.0105513)
Supplement: Figure S3 — Reproduction across a range of chronic stress temperatures (18 hours of heat stress). Experiments were performed exactly as described in Figure 2, except the duration of the heat stress was 18, not 24 hours. (PDF) [file pone.0105513.s003.pdf]

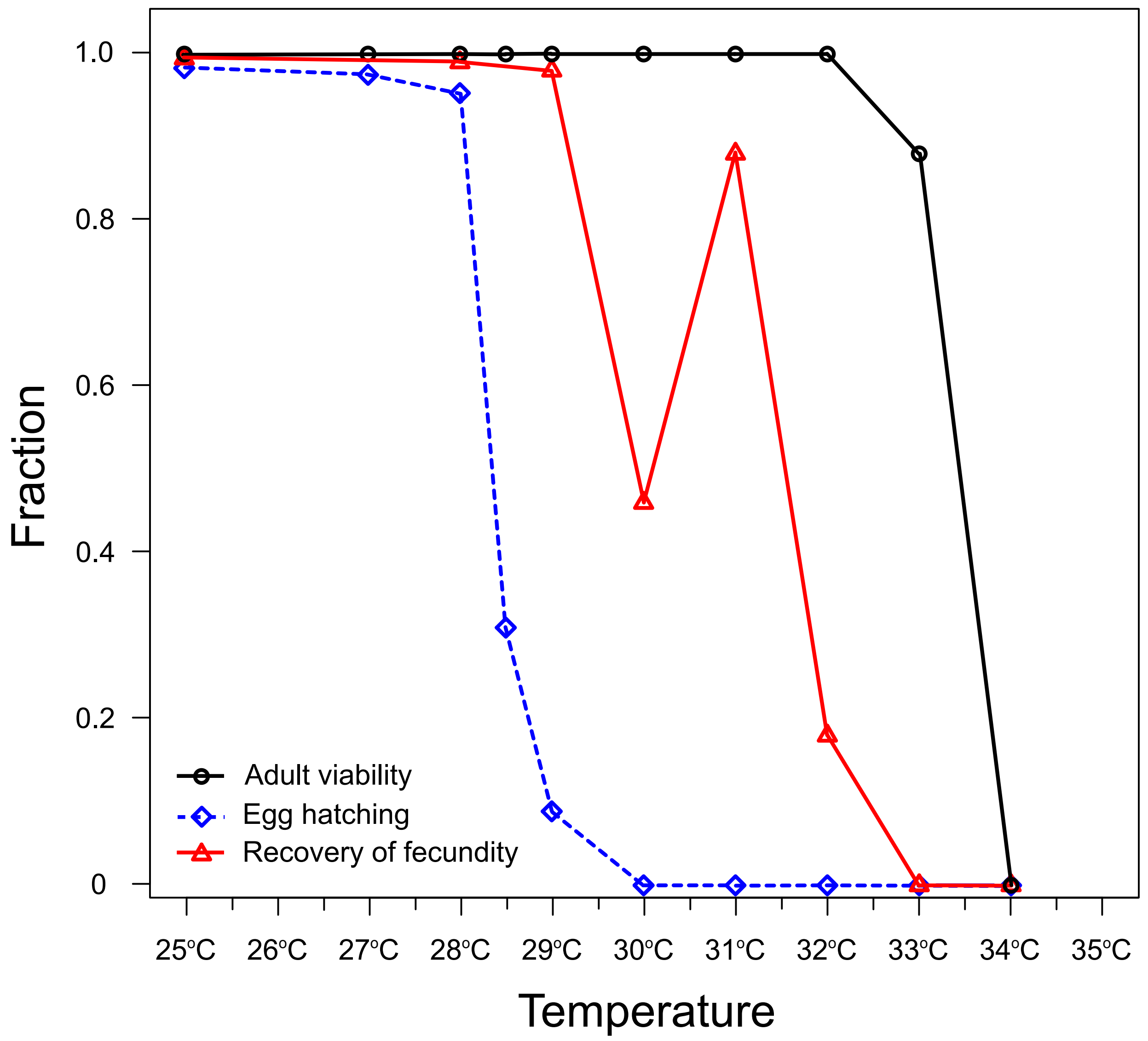

Figure S3. Reproduction across a range of chronic stress temperatures (18 hours of heat stress). Experiments were performed exactly as described in Figure 2, except the duration of the heat stress was 18, not 24 hours.
