# Supplementary material for: Balanced Trade-Offs between Alternative Strategies Shape the Response of C. elegans Reproduction to Chronic Heat Stress
Source: PLoS One. 2014 Aug 28;9(8):e105513. doi: 10.1371/journal.pone.0105513 (PMC4148340; doi:10.1371/journal.pone.0105513)
Supplement: Figure S4 — Census of oocytes in the proximal gonad and embryos in the uterus for worms kept at 20°C. Oocyte production in the anterior gonad arm precedes production in the posterior arm by about an hour. Error bars are s.d. (PDF) [file pone.0105513.s004.pdf]

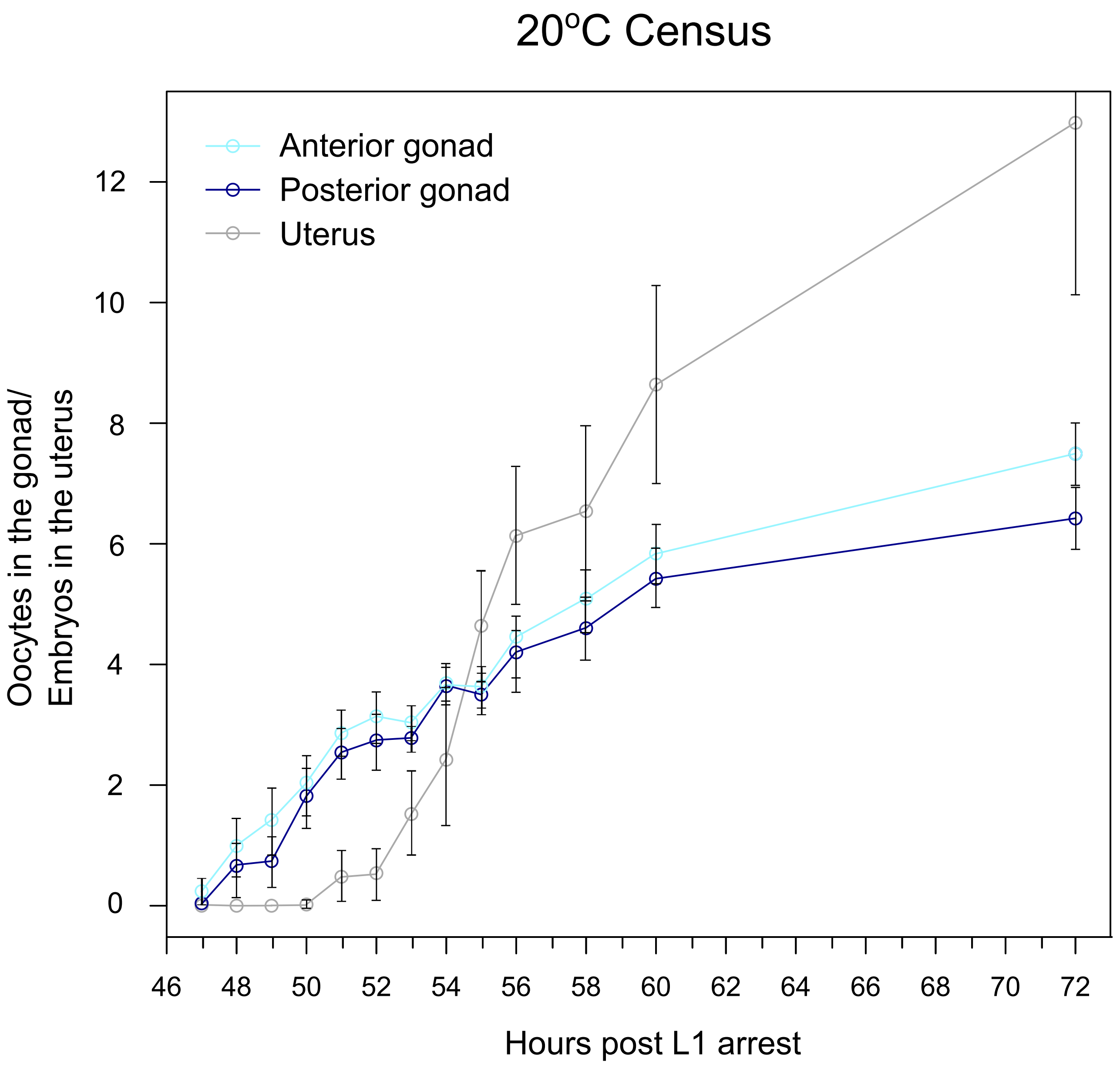

Figure S4. Census of oocytes in the proximal gonad and embryos in the uterus for worms kept at 20°C. Oocyte production in the anterior gonad arm precedes production in the posterior arm by about an hour. Error bars are s.d.
