# Supplementary material for: Balanced Trade-Offs between Alternative Strategies Shape the Response of C. elegans Reproduction to Chronic Heat Stress
Source: PLoS One. 2014 Aug 28;9(8):e105513. doi: 10.1371/journal.pone.0105513 (PMC4148340; doi:10.1371/journal.pone.0105513)
Supplement: Figure S8 — Spermatid transit at 20°C. The distribution of the mCherry signal between the three compartments - proximal gonad, spermatheca, and uterus – of both the anterior and posterior gonad arms. Error bars are s.d. (PDF) [file pone.0105513.s008.pdf]

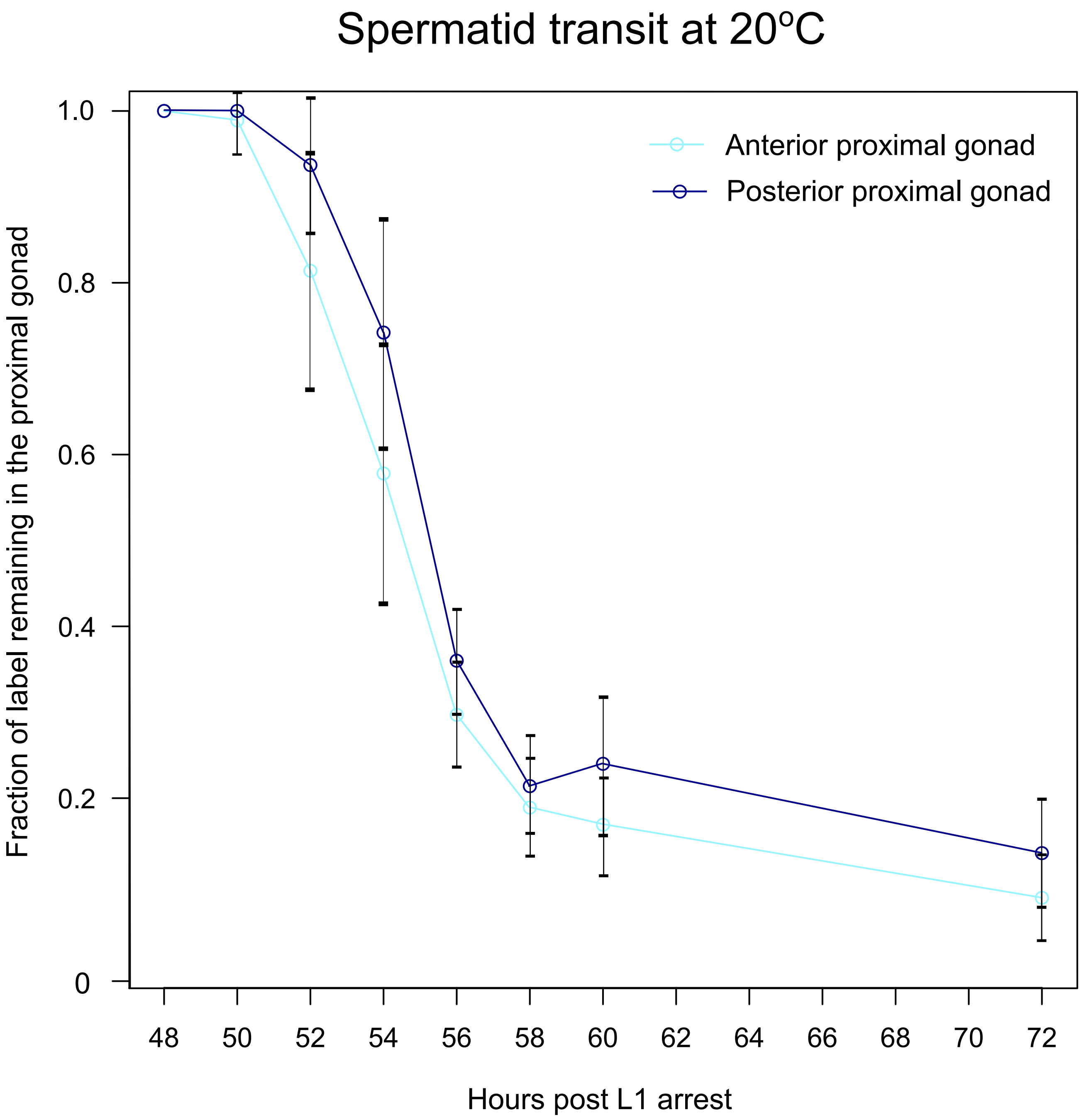


**Figure S8. Spermatid transit at 20°C.** The distribution of the mCherry signal between the three compartments - proximal gonad, spermatheca, and uterus – of both the anterior and posterior gonad arms. Error bars are s.d.
