# Supplementary material for: Balanced Trade-Offs between Alternative Strategies Shape the Response of C. elegans Reproduction to Chronic Heat Stress
Source: PLoS One. 2014 Aug 28;9(8):e105513. doi: 10.1371/journal.pone.0105513 (PMC4148340; doi:10.1371/journal.pone.0105513)
Supplement: Figure S9 — Schematic depiction of the reproductive system of C. elegans hermaphrodite after the first ovulation. The first ovulation typically occurs in the anterior gonad (left). The passage of the oocyte pushes some spermatids into the spermatheca where they become mature sperm capable of amoeboid movement. The oocyte is fertilized in the spermatheca and passes to the uterus. The posterior gonad (right) is shown prior to the first ovulation. Immature spermatids are concentrated in the proximal gonad. (PDF) [file pone.0105513.s009.pdf]

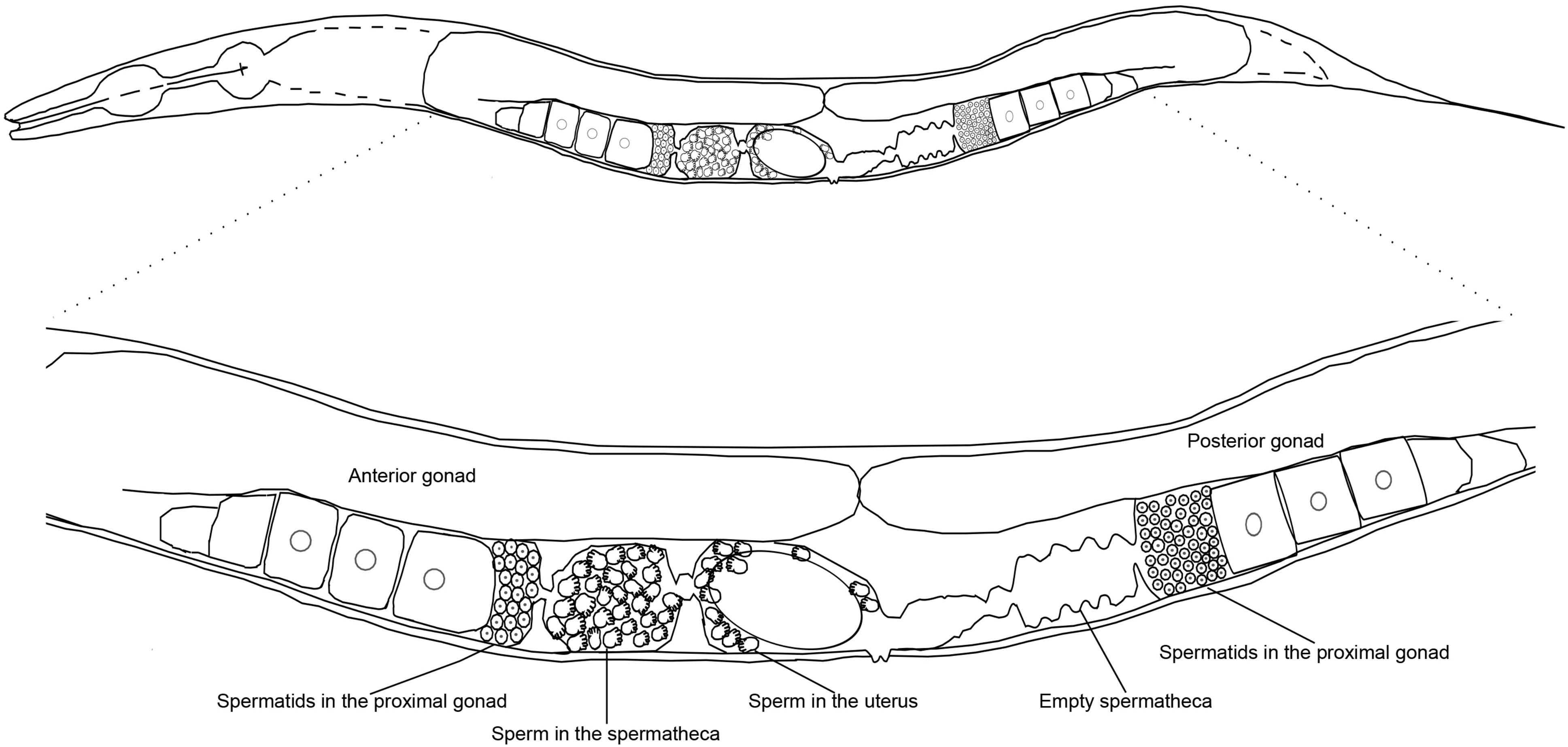


**Figure S9. Schematic depiction of the reproductive system of *C. elegans* hermaphrodite after the first ovulation.** The first ovulation typically occurs in the anterior gonad (left). The passage of the oocyte pushes some spermatids into the spermatheca where they become mature sperm capable of ameboid movement. The oocyte is fertilized in the spermatheca and passes to the uterus. The posterior gonad (right) is shown prior to the first ovulation. Immature spermatids are concentrated in the proximal gonad.
