# Supplementary material for: Balanced Trade-Offs between Alternative Strategies Shape the Response of C. elegans Reproduction to Chronic Heat Stress
Source: PLoS One. 2014 Aug 28;9(8):e105513. doi: 10.1371/journal.pone.0105513 (PMC4148340; doi:10.1371/journal.pone.0105513)
Supplement: Figure S11 — Spermatid transit for worms shifted to 30°C at 48 hours post L1 arrest. Error bars are s.d. (PDF) [file pone.0105513.s011.pdf]

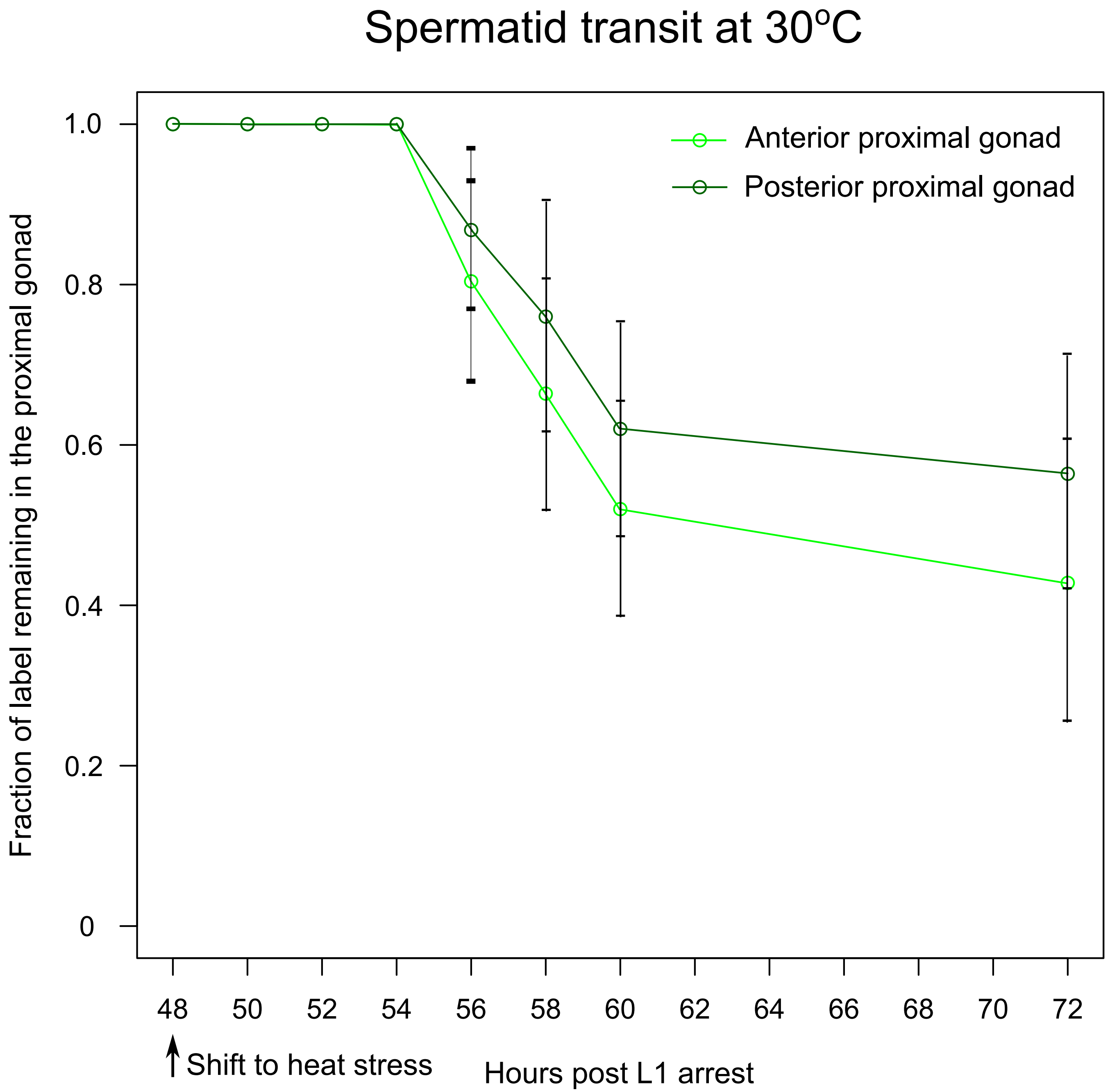

Figure S11. Spermatid transit for worms shifted to 30°C at 48 hours post L1 arrest. Error bars are s.d.
