# Supplementary figures and images for: Balanced Trade-Offs between Alternative Strategies Shape the Response of C. elegans Reproduction to Chronic Heat Stress
Source: PLoS One. 2014 Aug 28;9(8):e105513. doi: 10.1371/journal.pone.0105513 (PMC4148340; doi:10.1371/journal.pone.0105513)

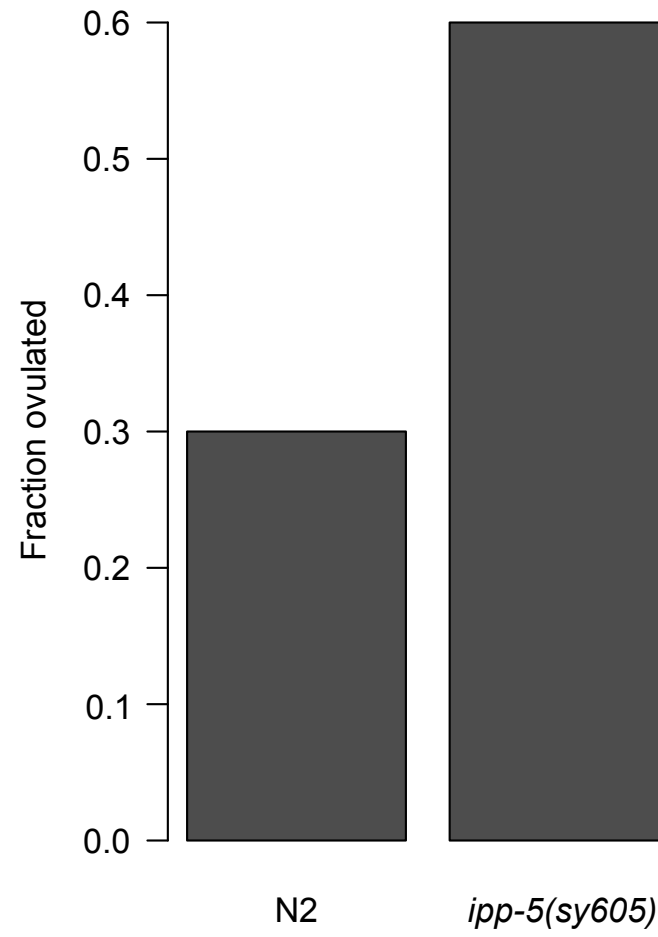

**Figure S13.** Fraction of N2 and *ipp-5(sy605)* worms that ovulated during 24 hours at 31°C.

Supplement: Figure S13 — Fraction of N2 and ipp-5(sy605) worms that ovulated at 31°C. (PDF) [file pone.0105513.s013.pdf]
