# Supplementary material for: Balanced Trade-Offs between Alternative Strategies Shape the Response of C. elegans Reproduction to Chronic Heat Stress
Source: PLoS One. 2014 Aug 28;9(8):e105513. doi: 10.1371/journal.pone.0105513 (PMC4148340; doi:10.1371/journal.pone.0105513)
Supplement: Figure S14 — Ovulation during heat stress (29°C) in wild type and mutant worms. The average number of embryos in the gonad (left) and eggs laid (right) for N2, itr-1(gf), crt-1 and N2 raised on 10 uM dantrolene. The total number of ovulations at 29°C can be obtained by adding values in each of these two categories. (PDF) [file pone.0105513.s014.pdf]

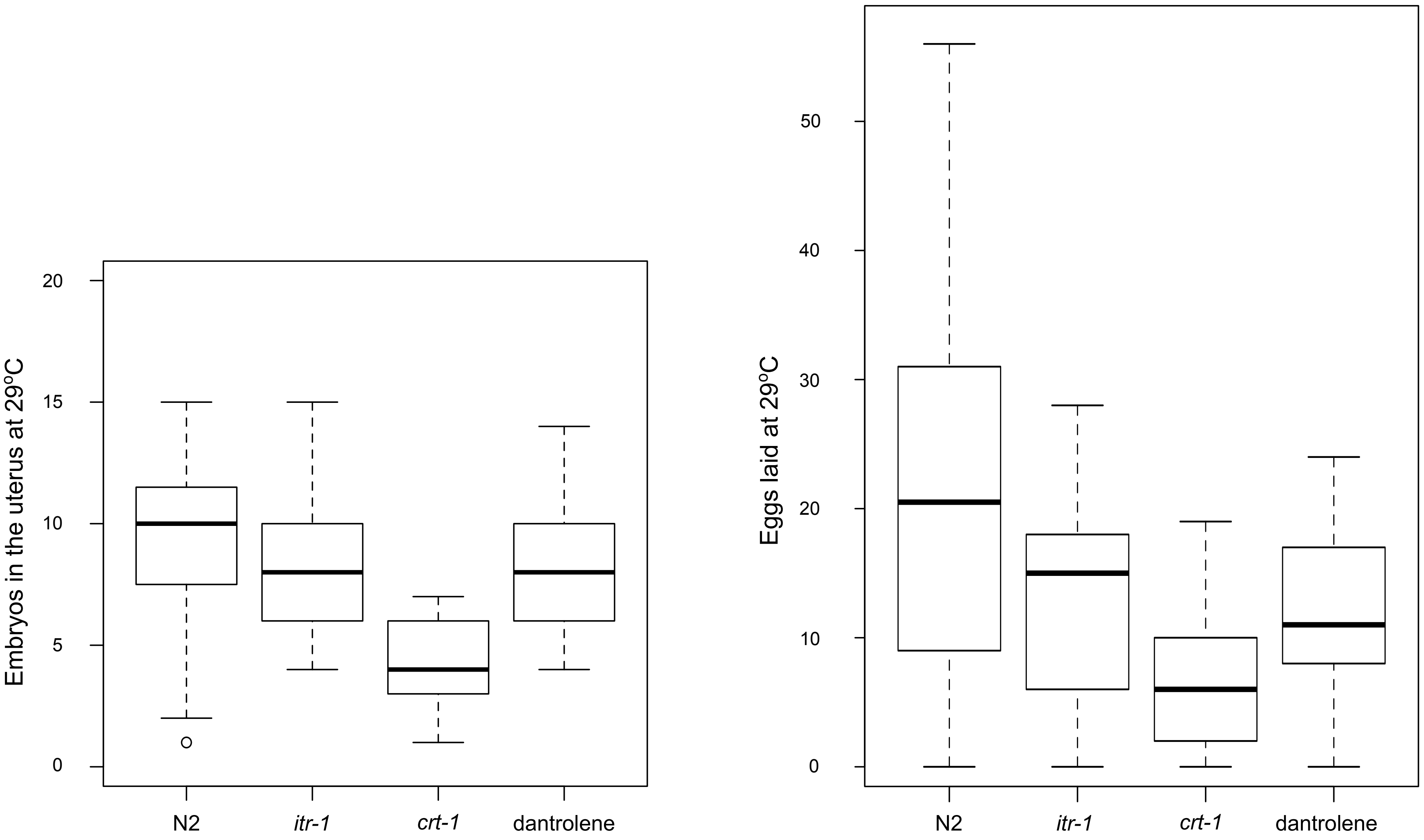

Figure S14. Ovulation during heat stress (29°C) in wild type and mutant worms. The average number of embryos in the gonad (left) and eggs laid (right) for N2, *itr-1(gf)*, *crt-1* and N2 raised on 10 uM dantrolene. The total number of ovulations at 29°C can be obtained by adding values in each of these two categories.
