# Supplementary material for: Balanced Trade-Offs between Alternative Strategies Shape the Response of C. elegans Reproduction to Chronic Heat Stress
Source: PLoS One. 2014 Aug 28;9(8):e105513. doi: 10.1371/journal.pone.0105513 (PMC4148340; doi:10.1371/journal.pone.0105513)

Figure S15. Timing of three milestones in the development of young adult hermaphrodites at 20ºC. Fraction of worms that have (A) produced the first oocyte, (B) begun to ovulate and produced the first embryo in the uterus. At each time point, multiple worms were observed. Fractions, not timing of events in individual animals are shown because once observed, animals could not be easily recovered. In contrast, (C) shows timing of first egg lay among 100 individually tracked worms.

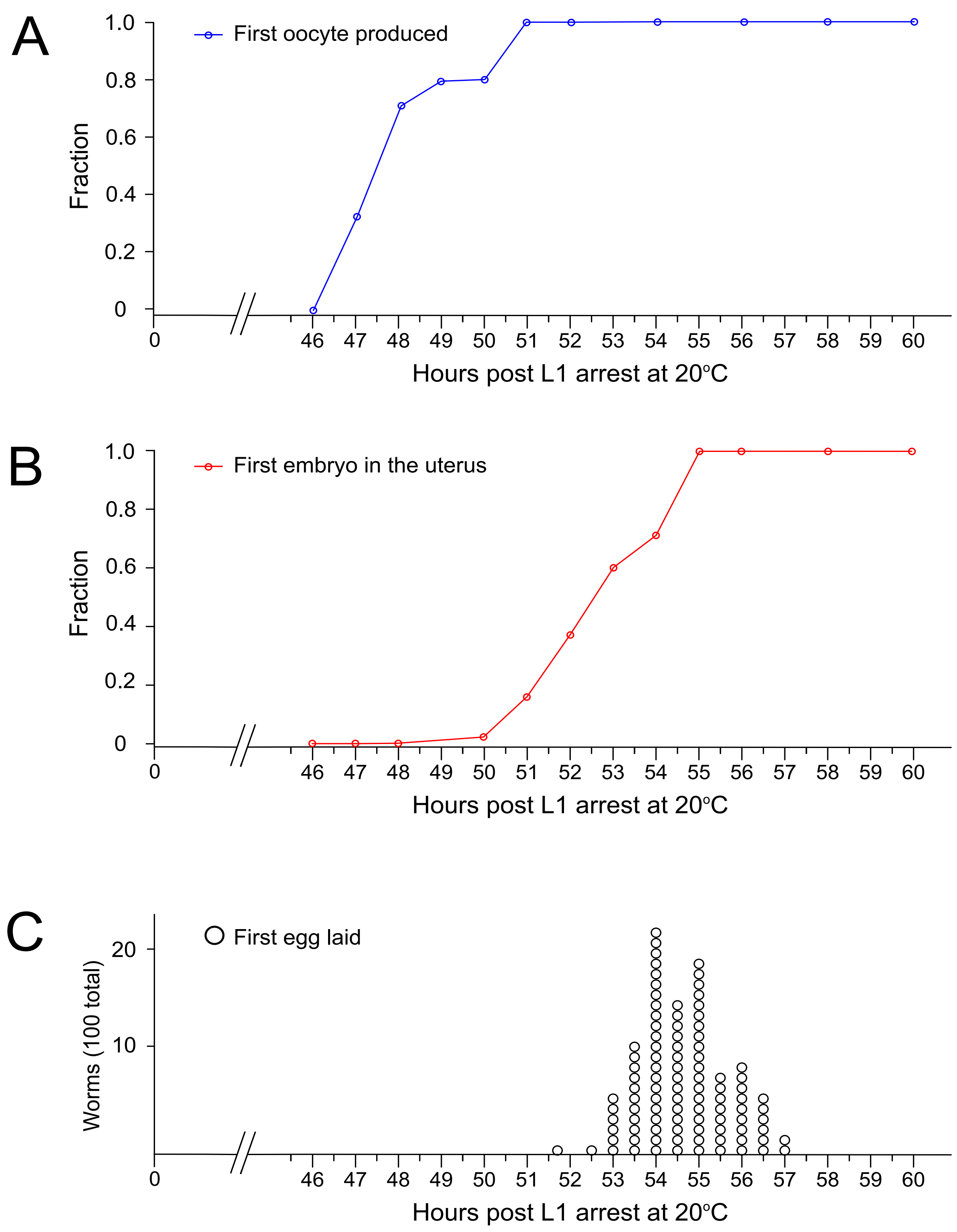

Supplement: Figure S15 — Timing of three milestones in the development of young adult hermaphrodites at 20°C. Fraction of worms that have (A) produced the first oocyte, (B) begun to ovulate and produced the first embryo in the uterus. At each time point, multiple worms were observed. Fractions, not timing of events in individual animals are shown because once observed, animals could not be easily recovered. In contrast, (C) shows timing of first egg lay among 100 individually tracked worms. (PDF) [file pone.0105513.s015.pdf]
