# Supplementary material for: Balanced Trade-Offs between Alternative Strategies Shape the Response of C. elegans Reproduction to Chronic Heat Stress
Source: PLoS One. 2014 Aug 28;9(8):e105513. doi: 10.1371/journal.pone.0105513 (PMC4148340; doi:10.1371/journal.pone.0105513)
Supplement: Table S1 — Summary of results on recovery after heat stress. (PDF) [file pone.0105513.s016.pdf]

Table S1. Summary of results on recovery after heat stress.

Duration		28°C	29°C	30°C	30.5°C	31°C	31.5°C	32°C	33°C
12 Hours	Fraction recovered	-	1.00	0.96	-	1.00	-	0.94	0.92
12 Hours	Average Brood size	-	41.1	19.9	-	44.8	-	17.3	3.3
18 Hours	Fraction recovered	1.00	0.94	0.46	-	0.88	-	0.40	0.28
18 Hours	Average brood size	98.3	7.1	1.0	-	3.1	-	1.3	0.5
24 Hours	Fraction recovered	0.88	0.12	0.10	0.10	0.58	0.32	0	0
24 Hours	Average brood size	2.6	0.5	0.40	0.3	1.8	0.9	0	0
36 Hours	Fraction recovered	-	0	0	-	0	-	0	0
36 Hours	Average brood size	-	0	0	-	0	-	0	0
