# Supplementary material for: Balanced Trade-Offs between Alternative Strategies Shape the Response of C. elegans Reproduction to Chronic Heat Stress
Source: PLoS One. 2014 Aug 28;9(8):e105513. doi: 10.1371/journal.pone.0105513 (PMC4148340; doi:10.1371/journal.pone.0105513)
Supplement: Table S3 — Mutants of the inositol 1,4,5-trisphosphate Ca2+ signaling pathway. (PDF) [file pone.0105513.s018.pdf]

Table S3. Mutants of the inositol 1,4,5-trisphosphate Ca^{2+} signaling pathway.

	Strain	Genotype	Phenotype
Used in study	PS3653	*ipp-5(sy605)* X.<br>loss of function	Ovulates two oocytes at a time,<br>slightly slow growth
	PS2368	*itr-1(sy327) unc-24(e138)* IV.<br>gain of function	Slow growth
	KJ216	*crt-1(jh101)* V.<br>loss of function	Very slow growth
	N2 on dantrolene	wild type	Slightly slow growth
Not used in study	JT73	*itr-1(sa73)* IV.<br>loss of function	Slow growth,<br>Very low brood size<br>Ovulation defects
	PS2286	*unc-38(x20) lfe-2(sy326*) I.<br>loss of function	Ovulation defect shears oocytes
	PS4886	*plc-3(tm1340)/mIn1[mIs14 dpy-10(e128)]* II.<br>loss of function	Ovulation defect shears oocytes
	CB6614	*egl-8(e2917)* V.<br>loss of function	Low brood size,<br>Impossible to synchronize
	MT1083	*egl-8(n488)* V.<br>loss of function	Low brood size,<br>Impossible to synchronize
