# Supplementary material for: Balanced Trade-Offs between Alternative Strategies Shape the Response of C. elegans Reproduction to Chronic Heat Stress
Source: PLoS One. 2014 Aug 28;9(8):e105513. doi: 10.1371/journal.pone.0105513 (PMC4148340; doi:10.1371/journal.pone.0105513)
Supplement: Table S4 — Developmental staging of ovulation mutants. The age at temperature shift reflects the degree that pleiotropic effects of mutations delayed development compared to wild type. (PDF) [file pone.0105513.s019.pdf]

Table S4. Developmental staging of ovulation mutants. The age at temperature shift reflects the degree that pleiotropic effects of mutations delayed development compared to wild type.

Strain	Average number of oocytes in the gonad	Average number of embryos in the uterus	Hours post hatch at shift
N2	1.65	0	48
N2 on dantrolene	1.80	0	52
KJ216	1.52	0.04	72
PS2286	1.52	0	51
PS2368	2.21	0.04	60
PS3653	1.60	0	52
