# Supplementary material for: Balanced Trade-Offs between Alternative Strategies Shape the Response of C. elegans Reproduction to Chronic Heat Stress
Source: PLoS One. 2014 Aug 28;9(8):e105513. doi: 10.1371/journal.pone.0105513 (PMC4148340; doi:10.1371/journal.pone.0105513)
Supplement: Table S5 — Timeline for a 24-hour heat stress experiment. (PDF) [file pone.0105513.s020.pdf]

Table S5. Timeline for a 24 hour heat stress experiment.

	Day	Worm preparation	Media preparation
	1	Chunk four plates with worms.	Pour NGM plates.
	2		5:00pm Inoculate fresh overnight culture of OP50.
	3	4:00pm Prepare synchronized culture of N2.	5:00pm Seed six lawn plates with OP50.
	4	10:00am Plate synchronized culture onto lawn plates. Incubate at 20°C.	
	5		5:00pm Seed 50 plates with 5uL 1:1000 dilution of OP50.
	6	9:00am Single and stage N2<br>10:00 am Shift to heat stress temperature. Reset recording thermometer.	
24 hours heat stress	7	10:00am Shift worms back to 20°C.<br>Record minimum and maximum temperatures.<br>Count number of eggs laid at temperature.	
24 hours recovery	8	Count eggs on plate.	
48 hours recovery	9	Count larvae on plate.	
72 hours recovery	10	Count larvae on plate.	
96 hours recovery	11	Count larvae on plate.	
120 hours recovery	12	Count larvae on plate.	
