# Supplementary material for: Balanced Trade-Offs between Alternative Strategies Shape the Response of C. elegans Reproduction to Chronic Heat Stress
Source: PLoS One. 2014 Aug 28;9(8):e105513. doi: 10.1371/journal.pone.0105513 (PMC4148340; doi:10.1371/journal.pone.0105513)
Supplement: Figure S1 — Recovery of fecundity following 24 hours of stress at temperatures around 31°C. Experiments were performed as described in Figure 1E. Numbers of worms for each temperature: 30°C (100), 30.5°C (50), 31°C (100), 31.5°C (50), 32°C (50). (PDF) [file pone.0105513.s001.pdf]

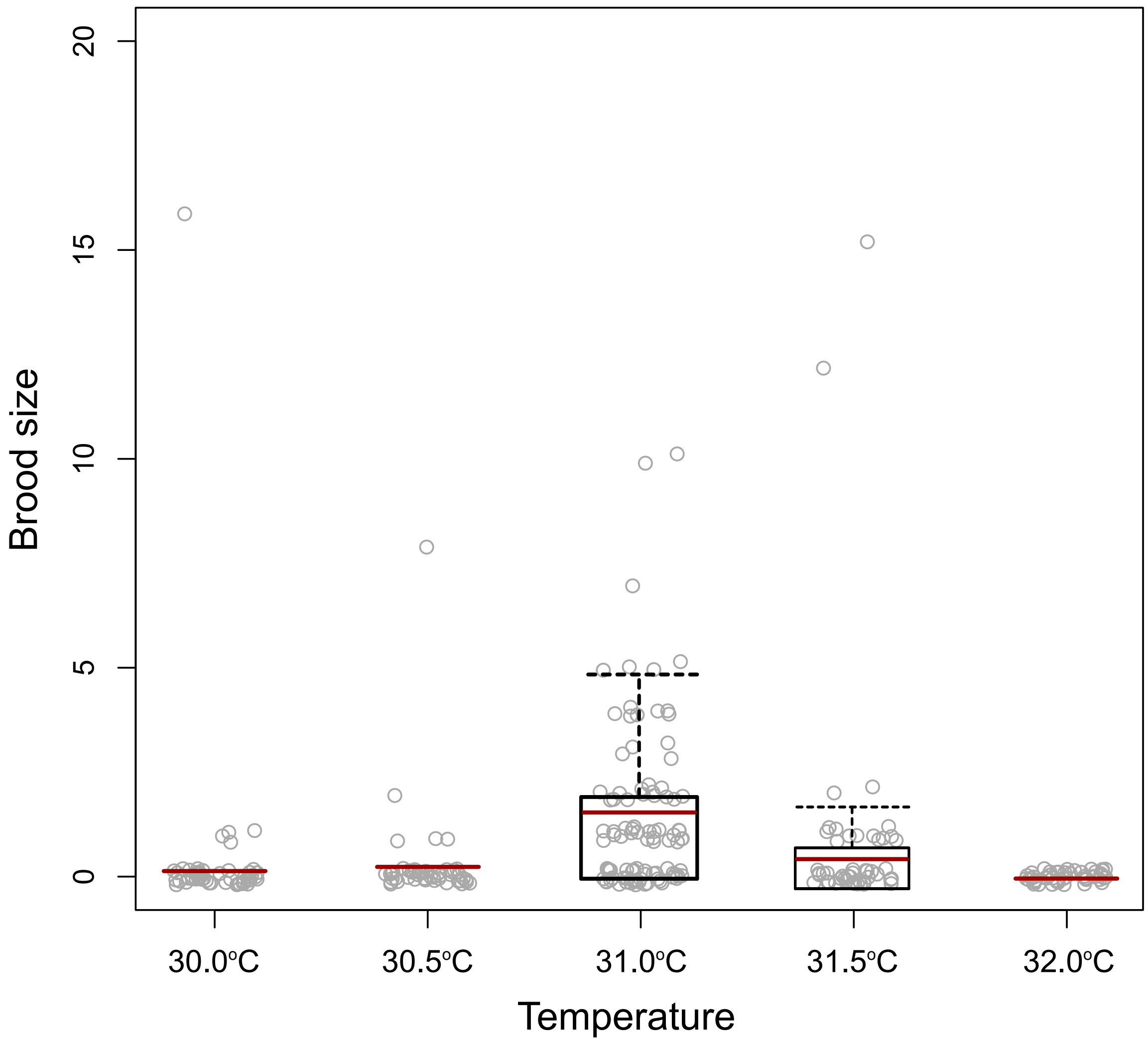

Figure S1. Recovery of fecundity following 24 hours of stress at temperatures around 31°C. Experiments were performed as described in Figure 1E. Numbers of worms for each temperature: 30°C (100), 30.5^{o}C (50), 31°C (100), 31.5^{o}C (50), 32°C (50).
